# Does economic growth always improve residents' dietary nutrition? Long-run relationships and phase differences in China from 1961 to 2018

**DOI:** 10.3389/fnut.2026.1835693

**Published:** 2026-06-12

**Authors:** Junda Shen, Yichuan Zeng

**Affiliations:** 1 International College Beijing, China Agricultural University, Beijing, China; 2 Department of Economics, University of Colorado Denver, Denver, CO, United States; 3 College of Economics and Management, China Agricultural University, Beijing, China

**Keywords:** calcium, China, dietary nutrition, economic growth, protein

## Abstract

**Background:**

China has experienced sustained economic growth alongside major changes in agricultural institutions, food circulation, and market openness. Whether economic growth improves residents' dietary nutrition under these changing conditions remains unclear.

**Methods:**

We use annual data for China from 1961 to 2018 to examine the long-run relationship between gross domestic product growth and residents' dietary nutrition. We employ autoregressive distributed lag models to identify the long-run relationship in the full sample, and then conduct segmented regressions based on major historical phases of China's economic development.

**Results:**

In the full sample, economic growth is positively associated with both nutritional quantity and nutritional quality in the long run, but this relationship is not stable over time. Before 1978, the relationship between economic growth and nutritional quantity in China is not significant, whereas after 1978 it becomes significantly positive. The relationship between economic growth and nutritional quality is significantly negative before 1978 and turns significantly positive thereafter. The relationship between economic growth and nutritional outcomes is clearly phase-specific, and its patterns differ between nutritional quantity and nutritional quality.

**Conclusions:**

Economic growth does not translate into nutritional improvement in a uniform way. In China, the relationship between economic growth and nutritional outcomes depends on the historical phase and differs between nutritional quantity and nutritional quality. Understanding the nutritional consequences of economic growth requires placing them in the broader context of institutional transformation and the evolution of the food system.

## 1 Introduction

Over the past several decades, China has achieved rapid economic growth, while its agricultural institutions and food market environment have undergone profound changes (1–4). Whether residents' dietary nutrition can improve alongside economic development is not only a question of nutrient adequacy, but also one of how the gains from development translate into improvements in residents' wellbeing.

Intuitively, economic growth should lead to better nutritional outcomes. Yet in the context of continuing changes in the institutional environment and the food system, this relationship may not operate in the same way across different historical phases. We still do not know whether the relationship between economic growth and nutritional outcomes is phase-specific, or whether such phase heterogeneity differs between nutritional quantity and nutritional quality. To address this question, we relate China's long-run process of economic development to nutritional outcomes and examine how economic growth is associated with residents' dietary nutrition across different historical phases.

Existing studies have offered several explanations for how economic growth shapes nutritional outcomes. First, income growth relaxes household budget constraints and enables greater consumption of higher-quality foods, thereby improving nutrient intake (5, 6). Using three waves of the China Health and Nutrition Survey data for 1997, 2004, and 2011, Gao et al. find that, during China's rapid economic growth, improvements in dietary quality during 1997–2004 mainly benefited high-income groups and widened income-related dietary inequality, whereas improvements during 2004–2011 became more evenly distributed but remained shaped by income and urban-rural differences (7). Evidence from low- and middle-income countries further shows that macroeconomic downturns and food price increases can weaken households' food purchasing capacity, reduce dietary diversity and increase the risks of child undernutrition and food insecurity (8, 9).

Second, improvements in domestic market development and food circulation conditions can alter food availability and price structures, thereby affecting residents' food demand and dietary patterns (10–13). Trade liberalization can also reshape the food supply and consumption environment, thereby influencing residents' dietary patterns and the path of nutrition transition (4, 14). Evidence from Eastern Africa shows that better market access is associated with higher food consumption expenditure, greater dietary diversity, and lower food insecurity among smallholder households (12).

These studies provide an important foundation for understanding the relationship between economic growth and dietary nutritional outcomes. However, most existing work focuses on average relationships within specific periods or on distributional differences across income groups, urban and rural populations, or countries. Less attention has been paid to whether the relationship between economic growth and nutritional outcomes itself changes across long historical phases. We argue that similar rates of economic growth may have different effects on nutritional outcomes across different stages of economic development and under different institutional environments. Furthermore, such phase differences may not manifest in the same way for nutritional quantity and nutritional quality.

From Acemoglu's institutional perspective, economic growth and its welfare consequences may exhibit phase differences under different institutional backgrounds (15). Major changes in agricultural organization, food circulation, and market openness can reshape how economic growth is translated into nutritional outcomes. We therefore adopt a phase-based framework to capture changes in institutional context. This allows us to examine whether the relationship between economic growth and nutritional outcomes remained stable over time or changed across phases.

Based on major turning points in China's economic development and institutional environment, we divide the 1961–2018 sample period into three phases. The first is the pre-reform period (1961–1978), when agricultural production was largely organized under a collective system, and grain circulation and distribution were centered on the state-led unified procurement and distribution system (16). The second is the reform and rural marketization period (1978–2001), when the household responsibility system, market-oriented circulation, and a higher degree of food market opening gradually changed the production, circulation, and consumption environment of food (1, 3). The third is the post-WTO integration period (2002–2018), which began after China joined the World Trade Organization (WTO) in 2001. In this phase, China became more deeply integrated into international agricultural and food markets, and the food supply, circulation, and consumption environment became more diversified.

We use annual data on gross domestic product (GDP) and residents' dietary nutrition in China to examine whether the relationship between economic growth and nutritional outcomes changes over the long course of history. We first use the Autoregressive Distributed Lag (ARDL) model to identify the long-run relationship in the full sample, and then combine it with segmented regressions to test whether this relationship remains stable across different historical phases. This empirical design allows us to identify the average long-run association and to further examine whether this association is phase-specific. Our results show that economic growth is positively associated with both nutritional quantity and nutritional quality in the full sample, but this relationship exhibits clear phase differentiation. We find that before 1978, the relationship between economic growth and nutritional quantity is not significant, while the relationship between economic growth and nutritional quality is negative. After 1978, both relationships turn positive.

Our main contributions are as follows. First, by examining the phase-specific relationship between economic growth and nutritional outcomes from a long-run historical perspective, we extend existing research, which has mainly focused on the average relationship between nutrition and income growth, market development, or trade liberalization within a given period. Second, by distinguishing between nutritional quantity and nutritional quality, we provide further empirical evidence that the relationship between economic growth and these two dimensions of nutrition may differ in both direction and magnitude. Finally, from the perspective of China's institutional and historical development, we highlight the boundary conditions of the relationship between economic growth and nutritional outcomes, thereby enriching the discussion of how institutional context shapes nutrition and health outcomes.

## 2 Materials and methods

### 2.1 Data sources and variable construction

This study uses annual data for China from 1961 to 2018. The definitions, measurements, and data sources of the variables are presented below.

#### 2.1.1 Economic development

Economic development is measured by the natural logarithm of real GDP per capita. The data are obtained from the World Bank. According to the World Bank definition, this indicator is calculated as gross domestic product at constant local currency prices divided by the midyear population and reflects average output per person (17).

#### 2.1.2 Nutritional quantity

Nutritional quantity is measured by the natural logarithm of annual per capita daily protein supply. The data are taken from Liu, Han, and Chai (18). Protein is commonly treated as a nutrient to encourage in diet quality assessment, and, relative to total calorie intake, it better reflects whether the intake of key nutrients has improved (14, 15).

#### 2.1.3 Nutritional quality

Nutritional quality is measured by calcium density as one micronutrient-density indicator. The data are also taken from Liu, Han, and Chai (18). Calcium is an essential micronutrient, and inadequate calcium intake has important public health implications (19). Following the nutrient density approach, we define calcium density as the amount of calcium supplied per 1,000 kcal in order to distinguish changes in dietary quality from increases in total calorie intake (20, 21).

#### 2.1.4 Urbanization rate

Urbanization rate is obtained from the World Bank and is defined as the share of the urban population in the total population (22). We include it as a contextual covariate to account for broad structural changes in agrifood systems, food access, and the dietary environment associated with long-run urbanization (23, 24).

### 2.2 Analysis strategies

First, we report descriptive statistics and plot trend figures and relationship graphs to present the basic patterns of the core variables over the sample period. Second, we conduct unit root tests to examine whether the variables are stationary and to assess whether the ARDL approach is appropriate. We use the Phillips-Perron (PP) test and the KPSS test to examine the time-series properties of the core variables (25, 26). The PP test takes the presence of a unit root as the null hypothesis, whereas the KPSS test takes stationarity as the null hypothesis. The ARDL framework is applied only after confirming that the variables do not exhibit clear I(2) characteristics (27).

Next, we estimate ARDL models using the full sample to examine whether a long-run relationship exists between economic growth and nutritional outcomes. For each outcome variable, we estimate both a baseline specification that includes only economic development and an extended specification that additionally includes the urbanization rate, and we further use the bounds test to determine whether cointegration exists (27). When a long-run relationship is supported, we further report the long-run coefficients and the error correction term (28).

In the ARDL analysis, the preferred specification for each outcome variable is determined according to four criteria. First, the bounds test must support cointegration. Second, the diagnostic results should be broadly acceptable. Third, the long-run coefficients and the error correction term should have clear economic meaning. Fourth, conditional on the above requirements, we prefer the more parsimonious model.

On the basis of the ARDL analysis, we further conduct segmented regressions. We estimate the coefficient on economic growth separately for the pre-reform period (1961–1978), the reform and rural marketization period (1979–2001), and the post-WTO integration period (2002–2018), in order to compare the relationship between economic growth and nutrition across phases. We estimate full-sample models with phase interaction terms and conduct pairwise F-tests for adjacent phases to examine whether the coefficient differences across phases are statistically significant.

All analyses were conducted in R, version 4.4.1 (R Foundation for Statistical Computing, Vienna, Austria).

## 3 Results

### 3.1 Descriptive statistics and long-run trends

**Figure 1:**
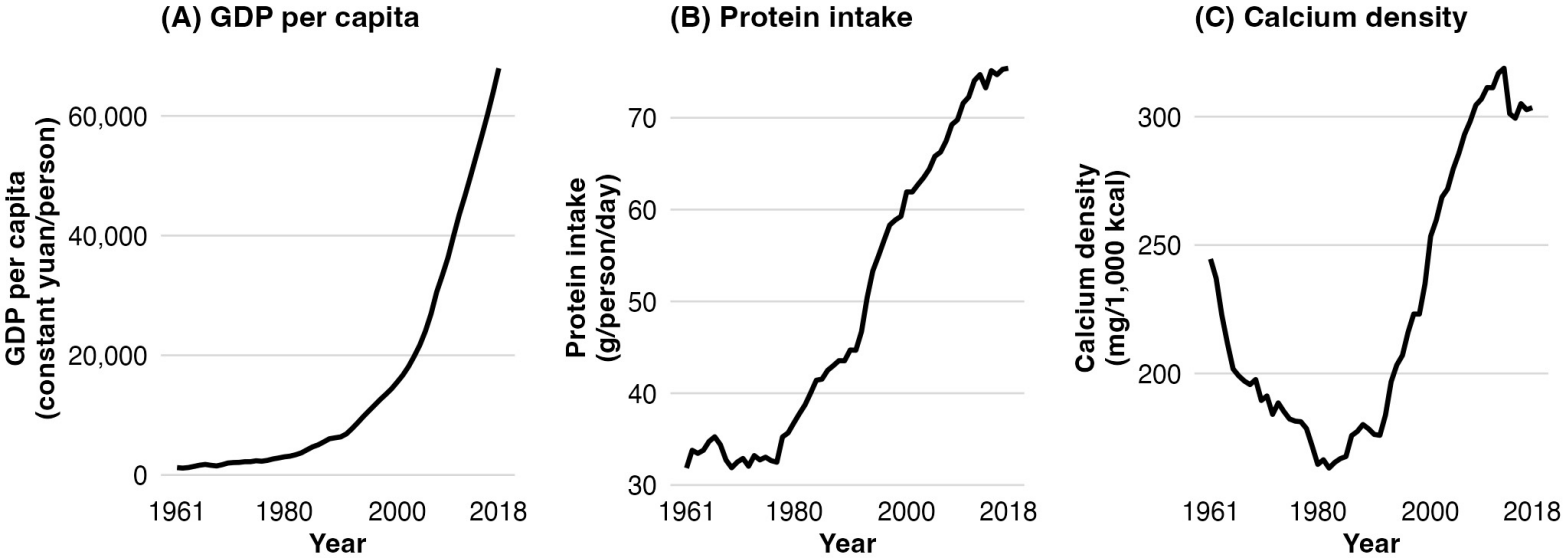
Long run trends in GDP per capita, protein intake, and calcium density. **(A)** GDP per capita. **(B)** Protein intake. **(C)** Calcium density.

Table 1 and Figure 1 jointly present the long-run changes in the core variables over the sample period. Overall, GDP increased continuously across the three phases, and the magnitude of this increase became much larger after 2000. Protein intake also showed an overall upward trend, with its phase mean rising from 33.263 g/person/day in 1961–1978 to 47.663 g/person/day in 1979–2001, and further to 70.325 g/person/day in 2002–2018. Figure 1B shows that protein intake changed little during 1961–1978. After 1978, it increased steadily. The pattern for calcium density was different. Its mean values were 198.295 and 192.656 mg/1,000 kcal in 1961–1978 and 1979–2001, respectively, and then increased to 298.740 mg/1,000 kcal in 2002–2018. Figure 1C shows a U-shaped pattern in calcium density. It declined from 1961 to around 1980 and then increased gradually. These results show clear phase differences in economic development, nutritional quantity, and nutritional quality.

**Table 1:** Descriptive statistics.

Variables	Phase	Mean	SD	Min	Max
GDP per capita	1961–1978	1871.220	457.842	1151.978	2678.456
	1979–2001	7927.949	4343.254	2843.601	16687.024
	2002–2018	40769.707	16121.285	18107.742	67962.409
Protein intake	1961–1978	33.263	1.074	31.850	35.258
	1979–2001	47.663	8.606	35.684	61.928
	2002–2018	70.325	4.511	62.722	75.393
Calcium density	1961–1978	198.295	19.195	178.556	244.584
	1979–2001	192.656	29.421	163.159	259.745
	2002–2018	298.740	14.638	268.648	318.843
Urbanization rate	1961–1978	17.602	0.553	16.836	19.294
	1979–2001	26.958	5.331	18.961	37.660
	2002–2018	50.132	7.167	39.090	61.500

(1) The sample includes 18 annual observations for 1961–1978, 23 for 1979–2001, and 17 for 2002–2018. (2) Units are constant Chinese yuan/person for GDP per capita, g/person/day for protein intake, and mg/1,000 kcal for calcium density.

**Figure 2:**
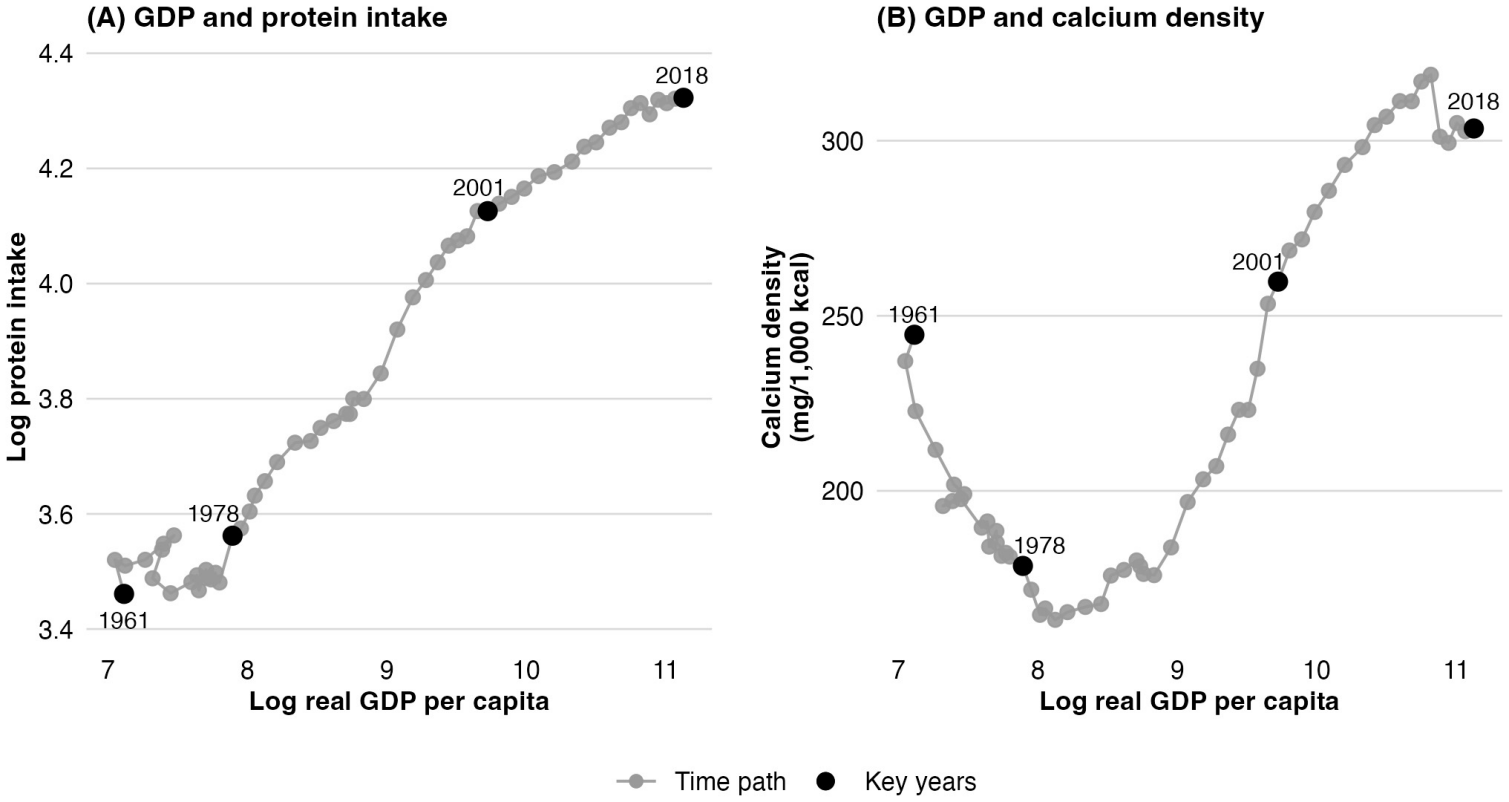
GDP and nutritional outcomes over time. **(A)** GDP and protein intake. **(B)** GDP and Calcium density.

Figure 2 shows clear phase differences in the relationship between GDP and nutritional outcomes. In Figure 2A, GDP and protein intake remained at relatively low levels and changed little during 1961–1978. After 1978, the two variables moved upward together, and their relationship became clearly positive. In Figure 2B, GDP and calcium density displayed a negative relationship from 1961 to around 1980. During the 1980s, the relationship turned positive, but the upward trend was relatively mild. From the 1990s onward, calcium density increased more steadily with GDP. A noticeable decline around 2013 further suggests that the relationship between GDP and calcium density was more complex and less stable than that between GDP and protein intake.

### 3.2 Unit root test results

As shown in Table 2, the unit root test results indicate that the four core variables can generally be treated as I(1). GDP and protein intake display relatively standard I(1) behavior. At levels, the PP statistics are insignificant, whereas the KPSS statistics are significant, indicating non-stationarity in levels. After first differencing, the PP statistics become significant, supporting stationarity in first differences. Calcium density and the urbanization rate show the same broad pattern. Although their KPSS statistics remain sensitive after first differencing, the PP results support stationarity in first differences. Overall, none of the variables shows clear evidence of I(2) behavior, so the ARDL approach is appropriate for the subsequent analysis (27).

**Table 2:** Results of unit root test.

Variables	Level		1st difference		Order
	PP	KPSS	PP	KPSS	
GDP per capita	−2.231	0.341^***^	−5.599^***^	0.462^*^	I(1)
Protein intake	−1.854	0.213^**^	−6.371^***^	0.189	I(1)
Calcium density	−2.950	0.353^***^	−4.170^***^	0.711^**^	I(1)
Urbanization rate	−2.039	0.380^***^	−4.618^***^	1.346^***^	I(1)

Significance levels: ^*^p < 0.10, ^**^p < 0.05, ^***^p < 0.01.

### 3.3 ARDL model results

Table 3 reports the ARDL estimates of the relationship between economic growth and nutritional outcomes. For each outcome variable, we estimate both a baseline model that includes only GDP per capita and an extended model that additionally includes the urbanization rate. Model 1 and Model 2 correspond to the baseline and extended specifications for the protein intake equation, respectively, while Model 3 and Model 4 correspond to the baseline and extended specifications for the calcium density equation.

**Table 3:** ARDL estimates of economic growth and nutrition outcomes.

Item	Protein intake		Calcium density	
	Model 1	Model 2	Model 3	Model 4
Long-run GDP effect	0.235	0.539	57.74	3632.64
Urbanization included		Yes		Yes
Bounds F-test	2.544	5.986^**^	14.901^***^	14.461^***^
ECT	−0.102^**^	−0.099^***^	−0.091^***^	−0.004^***^
Half-life	6.421	6.67	7.252	180.122
BG *p*-value	0.399	0.487	0.018	0.424
BP *p*-value	0.032	0.089	0.382	0.874

(1) Significance levels: ^*^p < 0.10, ^**^p < 0.05, ^***^p < 0.01. (2) Bounds *F*-test reports the ARDL bounds test for a level relationship. (3) Cointegration is judged primarily at the 5% significance level. (4) ECT denotes the error-correction term from the restricted ECM; a negative and significant ECT indicates convergence back to the long-run equilibrium. (5) Half-life is calculated from the estimated ECT and indicates the number of years required for half of a disequilibrium to dissipate.

The preferred specification is selected according to the four criteria discussed above: (1) whether cointegration is supported, (2) whether the diagnostic results are acceptable, (3) whether the long-run coefficient and the error-correction term have clear economic meaning, and (4) whether the model is as parsimonious as possible.

For protein intake, Model 2 is selected as the preferred specification. Model 1 does not pass the bounds test, with an F-statistic of 2.544, and therefore does not support a long-run cointegrating relationship. By contrast, Model 2 passes the bounds test once the urbanization rate is included. Its F-statistic is 5.986 and is significant at the 5% level, indicating a long-run relationship between economic growth and protein intake. In Model 2, the long-run coefficient on real GDP per capita is 0.539, while the error-correction term is −0.099 and significant at the 1% level.

The results from Model 2 indicate a positive long-run association between economic growth and protein intake. About 9.9% of the disequilibrium is corrected within one period. The implied half-life is approximately 6.67 periods. In terms of model diagnostics, the Breusch-Godfrey (BG) serial-correlation test *p*-value of Model 2 is 0.487, suggesting no clear evidence of serial correlation (29). Its Breusch-Pagan (BP) heteroskedasticity test *p*-value is 0.089, which is acceptable at the 5% level, although it may indicate weak borderline heteroskedasticity (30).

For calcium density, Model 3 is selected as the preferred specification. Both Model 3 and Model 4 pass the bounds test, indicating support for a long-run cointegrating relationship in both cases. In Model 3, the long-run coefficient on real GDP per capita is 57.74, while the error-correction term is −0.091 and significant at the 1% level. This indicates a positive long-run association between economic growth and improvements in calcium density. About 9.1% of the disequilibrium is corrected within one period. The implied half-life is approximately 7.252 periods. Although Model 4 also supports cointegration, its long-run GDP coefficient rises to 3632.64, while the error-correction term falls to −0.004, implying a half-life of 180.122 periods. The economic interpretation of Model 4 is therefore much weaker.

In terms of diagnostics, the BP *p*-value of Model 3 is 0.382, suggesting no clear heteroskedasticity problem, but its BG *p*-value is 0.018, indicating some evidence of serial correlation. By contrast, the BG and BP *p*-values of Model 4 are 0.424 and 0.874, respectively, indicating better diagnostic performance, but its long-run coefficient and adjustment speed are difficult to interpret economically.

Overall, the ARDL results indicate positive long-run associations between economic growth and improvements in both nutritional quantity and nutritional quality. At the same time, the diagnostic results suggest that some specifications may be subject to serial correlation or heteroskedasticity. Figures 1, 2 also show that the core variables do not follow a single and stable path over the sample period, and that both the direction and magnitude of change differ across phases. The parameter relationships captured by the full-sample ARDL models may therefore not remain constant throughout the entire period, and the residual diagnostic issues may be related to structural changes within the sample. In light of this, we further employ segmented regressions by dividing the sample according to major institutional turning points in China's economic development. This allows us to examine whether the relationship between economic growth and nutritional outcomes changes across phases and to further assess the issue of phase heterogeneity.

### 3.4 Segmented regression results

Table 4 reports the estimates from the segmented regressions. Overall, the relationship between economic growth and nutritional outcomes differs across phases, and this phase heterogeneity is evident for both nutritional quantity and nutritional quality.

**Table 4:** Phase-specific relationship between economic growth and nutrition outcomes.

Variables	Protein intake			Calcium density		
	1961–1978	1979–2001	2002–2018	1961–1978	1979–2001	2002–2018
GDP per capita	−0.000 (0.034)	0.314^***^ (0.053)	0.246^***^ (0.040)	−69.229^***^ (7.151)	48.025^***^ (4.408)	26.712^***^ (5.559)

(1) Significance levels: ^*^p < 0.10, ^**^p < 0.05, ^***^p < 0.01; (2) Standard errors are reported in parentheses.

For protein intake, the coefficient on GDP in 1961–1978 is 0.000 and statistically insignificant, indicating no clear relationship between economic growth and protein intake before the reform era. In 1979–2001, the GDP coefficient rises to 0.314 and is significant at the 1% level. In 2002–2018, the coefficient is 0.246 and remains significant at the 1% level. These results show that the positive association between economic growth and protein intake emerged mainly after 1978. The pairwise F-tests also support this conclusion. The coefficient difference between 1961–1978 and 1979–2001 is statistically significant (*F* = 50.545, *p* = 0.000), and the difference between 1979–2001 and 2002–2018 is also statistically significant (*F* = 19.323, *p* = 0.000).

For calcium density, the phase differences are more pronounced. In 1961–1978, the coefficient on ln GDP is −69.229 and is significant at the 1% level, indicating a significant negative association between economic growth and calcium density before the reform era. In 1979–2001, the coefficient turns positive at 48.025 and is significant at the 1% level. In 2002–2018, the coefficient is 26.712 and remains significant at the 1% level. This indicates that both the size and the sign of the relationship between economic growth and nutritional quality vary across phases. The pairwise F-tests further confirm this pattern. The coefficient difference between 1961–1978 and 1979–2001 is statistically significant (*F* = 134.309, *p* = 0.000), and the difference between 1979–2001 and 2002–2018 is also statistically significant (*F* = 9.484, *p* = 0.003).

Overall, the segmented regression results show clear phase heterogeneity in the relationship between economic growth and both nutritional quantity and nutritional quality. For protein intake, the relationship is insignificant before the reform era and significantly positive afterward. For calcium density, the relationship is significantly negative before the reform era and turns significantly positive afterward.

## 4 Discussion

### 4.1 Economic growth and nutritional outcomes under China's institutional transformation

Our results show that economic growth is positively associated with both protein intake and calcium density in the long run, but this relationship is phase-specific. For nutritional quantity, the positive association between economic growth and protein intake appears mainly after 1978. For nutritional quality, the relationship between economic growth and calcium density is significantly negative in 1961–1978, but turns significantly positive after 1978. These findings indicate that the institutional changes around China's reform era are crucial for understanding the relationship between economic growth and nutrition.

During 1961–1978, Chinese agriculture was largely organized under a collective production system, while grain circulation and distribution were centered on the state-led unified procurement and distribution system (1, 3). During this period, basic grain supply could be maintained, but the diversity of food types remained limited (31). The relationship between nutritional quantity and economic growth was not yet evident, while the negative association between nutritional quality and economic growth may reflect a dietary structure still centered on staple grains and a limited supply of higher-quality foods.

After 1978, China implemented the reform and opening-up policy and gradually introduced the household contract responsibility system with remuneration linked to output. These reforms strengthened incentives in agricultural production and contributed to agricultural growth (3). At the same time, the grain circulation and distribution system gradually shifted from a planned system to a more market-oriented one, allowing grain to be traded through markets (1). These institutional changes increased agricultural and food production capacity and improved the circulation of grain and agricultural products (11, 32). As a result, economic growth was more likely to translate through market allocation into improvements in both nutritional quantity and nutritional quality in residents' diets.

After joining the WTO in 2001, China became more deeply integrated into international agricultural and food markets, and the food supply and consumption environment became more diversified (4). Trade liberalization changed residents' diets and increased the consumption of animal-source foods, legumes, vegetables, and processed foods (14). In this phase, economic growth remained positively associated with both nutritional quantity and nutritional quality, but the coefficients were smaller than in the previous period. This suggests that, under conditions of greater openness and higher income, economic growth continued to improve nutritional quality, although its marginal effect weakened. One possible explanation is that the income elasticity of food quality declines as income rises (6). In other words, under conditions of greater openness and a more complex food system, economic growth still mattered, but its nutritional consequences might be shaped by how food was produced, circulated, and consumed.

Our findings have several practical implications for China and for other countries at similar stages of development. We suggest that the policy focus of nutrition improvement should vary across different stages of economic development. In the low-income stage, when the main nutrition challenge is still insufficient intake, the policy priority should be basic food security and adequate nutrient supply. In the stage of rising incomes and structural transition, the policy focus should shift toward improving dietary quality and access to more diverse foods. In the stage of higher income and more complex nutrition challenges, the policy focus should move further toward balanced diets, healthier eating patterns, and the joint goals of nutrition improvement, waste reduction, and lower environmental pressure. From this perspective, the nutritional consequences of economic growth are likely to depend on which nutrition challenges are most salient at a given stage of development.

### 4.2 Strengths and limitations

The main strength of our study is that we do not stop at the general question of whether economic growth improves nutrition. By distinguishing between nutritional quantity and nutritional quality, we identify more precisely how the relationship between economic growth and nutritional improvement differs across dimensions and further interpret this relationship in the context of China's institutional transformation. Our findings show that the relationship between economic growth and nutritional improvement is neither uniform nor linear, but varies across institutional contexts. In this way, we shed light on the historical process through which economic growth became associated with changes in residents' dietary nutrition.

At the same time, our study has several limitations. First, we identify long-run associations and their phase differences rather than the strict causal effect of any single policy shock. Although 1978 is an important institutional turning point, reform and opening up, the household responsibility system, adjustments in price and circulation systems, and broader structural changes all unfolded simultaneously. Our study design does not disentangle these mechanism one by one. Second, our measurement of nutritional outcomes remains partial. Protein intake captures one dimension of nutritional quantity, while calcium density is used only as one micronutrient-density indicator rather than as a comprehensive measure of overall dietary quality. Third, our study uses national annual average data, which are suitable for identifying aggregate long-run relationships, but do not allow us to examine population-level heterogeneity or more uneven patterns of nutritional change across urban and rural areas, regions, or households.

Given these limitations, there is substantial scope for future research. One important direction is to introduce provincial- or household-level data and exploit regional variation in reform exposure, market access, or trade liberalization to strengthen identification. Relatedly, future studies could use mediation or path-analysis frameworks with more suitable data and research designs to identify the causal mechanisms through which economic growth affects nutritional outcomes. Another direction is to broaden the set of nutritional indicators by incorporating dietary diversity, micronutrient adequacy, and indicators related to nutritional imbalance. In particular, more disaggregated and recent data would be better suited to examining issues such as overnutrition, nutritional imbalance, and the double burden of malnutrition in China's more recent development stage. More broadly, future research could place China's experience in a comparative global perspective. Global dietary quality remains moderate to low and varies substantially across countries, regions, education groups, age groups, and urban-rural populations (33), while global food-system transitions have improved the supply and affordability of nutritious diets without necessarily delivering better nutritional health, environmental sustainability, or social equity (34). Examining how these global patterns interact with national development stages would help clarify when economic growth translates into healthier and more equitable diets.

## 5 Conclusion

Based on data for China from 1961 to 2018, we find that economic growth is positively associated with improvements in nutritional outcomes in the long run, but this relationship shows clear phase heterogeneity. The positive association between economic growth and nutritional quantity appears mainly after 1978, whereas the relationship between economic growth and nutritional quality shifts from a significantly negative association before 1978 to a significantly positive association afterward. Economic growth does not translate into nutritional improvement in a linear way. Its effect may depend on boundary conditions such as the institutional environment and food circulation conditions. Understanding the historical process of nutritional improvement in China therefore requires placing economic growth in the broader context of institutional change and the evolution of the food system. This also suggests that nutrition policy should differ across stages of development, moving from basic food security, to dietary quality improvement, and then to balanced diets and healthier eating patterns as nutrition challenges become more complex.

## Data Availability

The original contributions presented in the study are included in the article/supplementary material, further inquiries can be directed to the corresponding author.

## References

[1] Sicular T. Plan and market in China's agricreultural commerce. J Polit Econ. (1988) 96:283–307. doi: 10.1086/261537

[2] Ash RF. The evolution of agricultural policy. China Q. (1988) 116:529–55. doi: 10.1017/S0305741000037887

[3] Lin JY. Rural reforms and agricultural growth in China. Am Econ Rev. (1992) 82:34–51.

[4] Ianchovichina E Martin W. Impacts of China's accession to the World Trade Organization. World Bank Econ Rev. (2004) 18:3–27. doi: 10.1093/wber/lhh030

[5] Hicks WW Johnson SR. Quantity and quality components for income elasticities of demand for food. Am J Agric Econ. (1968) 50:1512–7. doi: 10.2307/1237349

[6] Clements KW Si J. Engel's law, diet diversity, and the quality of food consumption. Am J Agric Econ. (2018) 100:1–22. doi: 10.1093/ajae/aax053

[7] Gao S Cuffey J Li G Li W. Diet in China during substantial economic growth: quality, inequality, trends, and determinants. China Econ Rev. (2024) 86:102208. doi: 10.1016/j.chieco.2024.102208

[8] Headey DD Ruel MT. Economic shocks predict increases in child wasting prevalence. Nat Commun. (2022) 13:2157. doi: 10.1038/s41467-022-29755-x 35444216 PMC9021262

[9] Headey D Ruel M. Food inflation and child undernutrition in low and middle income countries. Nat Commun. (2023) 14:5761. doi: 10.1038/s41467-023-41543-9 37717010 PMC10505228

[10] Huang J Rozelle S. Market development and food demand in rural China. China Econ Rev. (1998) 9:25–45. doi: 10.1016/S1043-951X(99)80002-9

[11] Park A Jin H Rozelle S Huang J. Market emergence and transition: arbitrage, transaction costs, and autarky in China's grain markets. Am J Agric Econ. (2002) 84:67–82. doi: 10.1111/1467-8276.00243

[12] Usman MA Haile MG. Market access, household dietary diversity and food security: evidence from Eastern Africa. Food Policy. (2022) 113:102374. doi: 10.1016/j.foodpol.2022.102374

[13] Choudhury S Bi AZ Medina-Lara A Morrish N Veettil PC. The rural food environment and its association with diet, nutrition status, and health outcomes in low-income and middle-income countries (LMICs): a systematic review. BMC Public Health. (2025) 25:994. doi: 10.1186/s12889-025-22098-4 40082817 PMC11907969

[14] Tian X Lin F. Trade liberalization and nutrition transition: evidence from China. Econ Hum Biol. (2023) 51:101304. doi: 10.1016/j.ehb.2023.101304 37716138

[15] Acemoglu D Johnson S Robinson JA. The colonial origins of comparative development: an empirical investigation. Am Econ Rev. (2001) 91:1369–401. doi: 10.1257/aer.91.5.1369

[16] Hong R Zhan M Wang F. What determines the development of a rural collective economy? A fuzzy set qualitative comparative analysis (fsQCA) approach. China Agric Econ Rev. (2023) 15:506–33. doi: 10.1108/CAER-12-2021-0244

[17] World Bank. GDP Per Capita (Constant LCU). World Development Indicators (n.d.). Available online at: https://databank.worldbank.org/metadataglossary/sustainable-development-goals-(sdgs)/series/NY.GDP.PCAP.KN (Accessed March 10, 2026).

[18] Liu A Han A Chai L. Assessing the nutrient adequacy in China's food supply from 1965 to 2018. Nutrients. (2021) 13:2734. doi: 10.3390/nu13082734 34444894 PMC8400167

[19] Passarelli S Free CM Shepon A Beal T Batis C Golden CD. Global estimation of dietary micronutrient inadequacies: a modelling analysis. Lancet Glob Heal. (2024) 12:e1590–9. doi: 10.1016/S2214-109X(24)00276-6 39218000 PMC11426101

[20] Nicklas TA Drewnowski A O'Neil CE. The nutrient density approach to healthy eating: challenges and opportunities. Public Health Nutr. (2014) 17:2626–36. doi: 10.1017/S136898001400158X 25166614 PMC10282407

[21] Drewnowski A Dwyer J King JC Weaver CM A. proposed nutrient density score that includes food groups and nutrients to better align with dietary guidance. Nutr Rev. (2019) 77:404–16. doi: 10.1093/nutrit/nuz002 31222368 PMC6489166

[22] World Bank. Urban Population (% of Total Population). World Development Indicators (n.d.). https://databank.worldbank.org/metadataglossary/world-development-indicators/series/SP.URB.TOTL.IN.ZS (Accessed March 10, 2026).

[23] Popkin BM. Urbanization, lifestyle changes and the nutrition transition. World Dev. (1999) 27:1905–16. doi: 10.1016/S0305-750X(99)00094-7

[24] Amare M Arndt C Abay KA Benson T. Urbanization and child nutritional outcomes. World Bank Econ Rev. (2020) 34:63–74. doi: 10.1596/36071

[25] Phillips PCB Perron P. Testing for a unit root in time series regression. Biometrika. (1988) 75:335–46. doi: 10.1093/biomet/75.2.335

[26] Kwiatkowski D Phillips PCB Schmidt P Shin Y. Testing the null hypothesis of stationarity against the alternative of a unit root: how sure are we that economic time series have a unit root? J Econom. (1992) 54:159–78. doi: 10.1016/0304-4076(92)90104-Y

[27] Pesaran MH Shin Y Smith RJ. Bounds testing approaches to the analysis of level relationships. J Appl Econom. (2001) 16:289–326. doi: 10.1002/jae.616

[28] Engle RF Granger CWJ. Co-integration and error correction: representation, estimation, and testing. Econometrica. (1987) 55:251–76. doi: 10.2307/1913236

[29] Godfrey LG. Testing for higher order serial correlation in regression equations when the regressors include lagged dependent variables. Econometrica. (1978) 46:1303–10. doi: 10.2307/1913830

[30] Breusch TS Pagan AR. A simple test for heteroscedasticity and random coefficient variation. Econometrica. (1979) 47:1287–94. doi: 10.2307/1911963

[31] Choudhury S Headey D. What drives diversification of national food supplies? A cross-country analysis. Glob Food Secur. (2017) 15:85–93. doi: 10.1016/j.gfs.2017.05.005 29276671 PMC5727671

[32] Rozelle S Park A Huang J Jin H. Liberalization and rural market integration in China. Am J Agric Econ. (1997) 79:635–42. doi: 10.2307/1244163

[33] Miller V Webb P Cudhea F Shi P Zhang J Reedy J . Global dietary quality in 185 countries from 1990 to 2018 show wide differences by nation, age, education, and urbanicity. Nat Food. (2022) 3:694–702. 37118151 10.1038/s43016-022-00594-9 PMC10277807

[34] Ambikapathi R Schneider KR Davis B Herrero M Winters P Fanzo JC. Global food systems transitions have enabled affordable diets but had less favourable outcomes for nutrition, environmental health, inclusion and equity. Nat Food. (2022) 3:764–79. doi: 10.1038/s43016-022-00588-7 37118149

